# Genotyping of 30 kinds of cutaneous human papillomaviruses by a multiplex microfluidic loop-mediated isothermal amplification and visual detection method

**DOI:** 10.1186/s12985-020-01373-3

**Published:** 2020-07-09

**Authors:** Yining Wang, Ge Ge, Rui Mao, Zhuo Wang, Yu-Zhe Sun, Yu-Guang Du, Xing-Hua Gao, Rui-Qun Qi, Hong-Duo Chen

**Affiliations:** 1 Department of Dermatology, The First Hospital of China Medical University, No.155 Nanjing Bei Street, Heping District, Shenyang, Liaoning Province 110001 PR China; 2 NHC Key Laboratory of Immunodermatology (China Medical University), No.155 Nanjing Bei Street, Heping District, Shenyang, Liaoning Province 110001 PR China; 3 Key Laboratory of Immunodermatology (China Medical University), Ministry of Education, No.155 Nanjing Bei Street, Heping District, Shenyang, Liaoning Province 110001 PR China; 4 Key Laboratory of Immunodermatology, Liaoning Province, No.155 Nanjing Bei Street, Heping District, Shenyang, Liaoning Province 110001 PR China; 5 Key Laboratory of Immunodermatology (China Medical University), Department of education of Liaoning Province, No.155 Nanjing Bei Street, Heping District, Shenyang, Liaoning Province 110001 PR China; 6 Key Laboratory of Biopharmaceutical Production & Formulation Engineering, PLA and State Key Laboratory of Biochemical Engineering, Institute of Process Engineering, Chinese Academy of Sciences, Beijing, 100190 PR China; 7 Dermatology Hospital of Southern Medical University, No. 2 Lujing Road, Yuexiu District, Guangzhou, Guangdong Province 510091 PR China

**Keywords:** HPV, Detection, LAMP, Microfluidic, Genotyping

## Abstract

**Background:**

Human papillomaviruses (HPVs), a group of non-enveloped small viruses with double-stranded circular DNA which lead to multiple skin diseases such as benign warts, are commonly seen in clinics. The current HPV detection systems aim mainly at mucosal HPVs, however, an efficient clinical approach for cutaneous HPVs detection is lacking.

**Objectives:**

To establish a rapid detection system for cutaneous HPVs using a colorimetric loop-mediated isothermal amplification (LAMP) with hydroxynaphthol blue (HNB) dye in combination with microfluidic technology.

**Methods:**

L1 DNA sequences of the 30 cutaneous HPVs were chemically synthesized, and LAMP primers against L1 DNA were designed with use of an online LAMP designing tool. Isothermal amplification was performed with use of a water bath and the amplification results were inspected with the naked eye. Using PCR sequencing as a control method, the specificity and sensitivity of the new detection system were obtained by detecting clinical samples.

**Results:**

The lower detection limit of the LAMP assay was 10^7^ viral DNA copies/μl when tested on synthesized L1 DNA sequences, which was better than the conventional PCR. Compared to PCR sequencing, the sensitivity of HPV27, HPV2, HPV1, HPV57, HPV3, HPV4, HPV7 and HPV75 genotypes detections were 100%, whereas the specificity was 34.55, 45.12, 95.83, 98.59 and 97.62% respectively, when tested on clinical samples.

**Conclusions:**

The new cutaneous type HPV detection system is characterized by both a good sensitivity and specificity compared to conventional methods.

## Introduction

Human papillomavirus (HPV) is a group of double-stranded circular DNA viruses with a genome of around 8000 base pairs. There have been currently over 200 HPV species identified, with new species being added following the latest research findings [1]. HPVs are mainly sorted into two types (cutaneous type and mucous type) according to the diseases they generate. Most cutaneous HPVs belong to the beta genus, with the others to alpha, gamma, mu and nu genera [2]. HPVs are associated with several skin diseases, such as benign skin warts [3], actinic keratosis (AKs) as well as non-melanoma skin cancers (NMSCs) [4]. Besides, HPVs are also often detected in healthy people [5]. Common, plantar and flat warts are the most common cutaneous warts, each with a different source and type of HPV infection. For example, common warts are usually due to HPV1, 2 and 4 infections, with HPV 2 being the most common [6], whereas flat warts are often caused by HPV 3, 10 and 28 infections. Clinical manifestations of cutaneous warts, such as the degree of keratosis, vary with different HPV infections [7]. Moreover, the prevalence of specific HPV infections is linked with geographical location. Thus, the species-specific detection of HPV infections combined with analysis of the treatment effects can provide the molecular epidemiological evidence for individualized treatment against HPV infections and improve therapeutic efficacy. Most HPV detection methods currently available are for mucosal HPVs, leaving the detection of cutaneous HPVs nonexistent within the clinical setting [8, 9].

Loop-mediated isothermal amplification (LAMP) is a technique that can amplify DNA under conditions of constant temperature. The reaction process involves binding of 4 sets of specific primers to 6 respective DNA sites along with an automatic strand displacement amplification catalyzed by Bst DNA polymerase [10]. Compared with conventional DNA amplification methods, this technology has obvious advantages, as it requires no high-tech equipment and can be rapidly performed at a lower cost. Therefore, it often serves as a useful analysis tool, especially in resource-derived areas [11]. The sensitivity of the LAMP method is not affected by the non-targeted DNA in the sample, making it more widely used than conventional PCR reactions [10, 12]. The LAMP reaction primers must simultaneously bind to the six specific sites of target DNA to start amplification, both the sensitivity and specificity of LAMP reactions are greater than that of conventional PCR reactions [13] as well as other detection methods [10, 13, 14]. In addition, during LAMP reactions, the consuming of dNTP substrates produces large numbers of pyrophosphate ions [15]. These pyrophosphate ions can then bind to magnesium ions in the buffer solution to form white precipitants [10, 13, 15, 16], resulting in changes which can be visually detected [17]. In order to obtain a more obvious colorimetric result, hydroxynaphthol blue (HNB) [18] can be added to the reaction buffer to generate a purple color. Once magnesium ions are precipitated by pyrophosphate, HNB then turns the reaction solution to a sky blue color [19], indicating for a positive amplification result. Given the convenience of these simple and reliable visual observations, the LAMP detection method is now widely applied [19–21].

The LAMP reaction has been widely used as a rapid and accurate nucleic acid amplification method in the detection of pathogens such as human immunodeficiency virus [22], Severe Acute Respiratory Syndrome Coronavirus [23], hepatitis B virus [24] and H5 avian influenza virus [25]. Mucosal HPV can cause cervical cancer and genital warts. The risk grading of cervical cancer by detecting HPV type is of great significance for guiding clinical treatment and prognosis. LAMP technology has also been used in the detection of mucosal HPV type. Cutaneous HPV testing has not been given enough attention. LAMP technology is convenient for clinical detection because of its convenient operation and low cost. It is of great significance to promote the development of cutaneous HPV genotyping.

Despite of the rapidness and accuracy of LAMP reaction, it is currently performed in normal polypropylene tubes requiring sample volumes of tens to hundreds of microliters. This factor not only limits further implementations of the technique, especially in cases where pathogens are present within minute amounts of sample, but also hinders its capacity to be established as a complete diagnostic system [26–28]. In addition, LAMP technology is prone to aerosol contamination because it can amplify target gene within a short time. Aerosol contamination can cause serious false positives. Therefore, it is clear that much work is needed to improve the LAMP reaction method in the field of pathogen detection [29].

Microfluidic technology, which has been applied in the lab-on-chip system, can process microliters of solution within a capillary channel, and is able to simultaneously perform reaction controls, detections and assessments results within a single chip. It provides a high-throughput, low-cost sealed reaction system [30]. By combining LAMP reactions with the microfluidic chip system, we obtained a new LAMP reaction system capable of reducing buffer volume and lowering false positive rates caused by aerosol contamination during pathogen detection, which broadened LAMP reaction’s potential for implementation as a novel pathogen detection system [31].

## Materials and methods

### HPV-L1 gene synthesis and plasmid extraction

L1 gene sequences of 30 types of HPV were searched and downloaded from the NCBI nucleotide database, and synthesized into a pUC57 plasmid with an ampicillin resistance with help of The Beijing Genomics Institute. The plasmids were then transfected into *E.coli* and screened through LB culture medium with 100 μg/ml ampicillin. HPV-L1 plasmids were extracted with use of the TIANprep Mini Plasmid Kit following the manufacturers’ instructions. Briefly, bacterial solution was centrifuged, after which the bacteria pellet was collected, suspended with RNase A-added P1 solution and lysed with solution P2. The plasmids were collected by centrifugation. The plasmid elutions were quantified with use of UV-Vis Spectrophotometer Q500 (Quawell).

### Clinical diagnosis and sample collection

Samples of scrapes from the skin of out-patients diagnosed with skin warts were obtained from the Department of Dermatovenorology at the First Hospital of China Medical University. All patients were instructed as to the purpose of the study and signed informed consent forms. All diagnoses met the clinical criteria for skin warts, and patients were diagnosed with either common warts, plantar warts or verruca plana, and assigned to specific groups as based upon their diagnosis.

### LAMP primers design and groups

HPV-specific LAMP primers were designed with use of an online LAMP primer design website (http://primerexplorer.jp/e/). The designing algorithm was established with the lengths of the primers being 15–22 bp for F1c/B1c and 15–20 bp for F2/B2 and F3/B3. The GC ratio was set to 40–60% and the remaining parameters were all set to default values. The DNA sequences of all primers are shown in Supplementary Table 1. All primers were synthesized with help of the Sangon Co. Ltd. (Shanghai, China).

All 30 species of HPVs were assigned into one of 3 major groups according to their viral characteristic types (low-risk, high-risk and rare). The low-risk group contained 18 HPV species, which were then divided into three sub-groups (Sub-group 1 contains HPV1,2,4,7,10, and 57; Sub-group 2 contains HPV3,27,41,49,65, and 75; Sub-group 3 contains HPV28,29,76,77,117, and 125). There were also 6 HPV species within the high-risk and rare groups each (High-risk group contains HPV5,8,12,14,48, and 50; Rare group contains HPV9,63,94,95,115, and 160). Specific LAMP primers for each group were independently identified and constructed onto the same specific microfluidic chip for further detections.

### LAMP reaction amplification using microfluidic chips

Microfluidic chips with 8 reaction wells were designed and manufactured by the State Key Laboratory of Biochemical Engineering, Institute of Process Engineering, Chinese Academy of Sciences. For microfluidic LAMP reaction amplifications, each group, together with one or two negative control samples and a positive control sample (8 samples in all), were mounted onto one microfluidic chip. The positive control and tested HPV primers were added into the reaction well on the microfluidic chip, whereas the negative control well remained empty for the presence or absence of aerosol contamination. Specificity test was performed individually within each HPV group. As the low-risk group contained three sub-groups, the primers assigned were based on principles to obtain maximal degrees of specificity across different sub-groups while ensuring 100% specificity within each sub-group. In this way, cross-reactions among the three sub-groups were maintained at minimal levels. The LAMP reaction buffer was prepared by mixing 4 μl of Bst DNA polymerase (8 U), 3 μl of HNB (3 mM), 37.5 μl of 10 × ThermoPol reaction buffer (1.4 μM dNTP, 10 mM KCl, 10 mM (NH_4_)_2_SO_4_, 20 mM Tris-HCl, 0.1%Tween 20, 0.8 M betaine, 8 mM MgSO_4_), 1 ng of respective HPV-L1 plasmid, and nuclease-free water to obtain a final volume of 75 μl. A total volume of 75 μl LAMP primer-free reaction buffer was loaded onto the microfluidic chips that had been pre-treated with respective HPV primers in each reaction well (1.6 μM for each of the inner primers - FIP and BIP and 0.2 μM for each of the outer primers - F3 and B3). The chips were then immersed into a 60 °C water bath (Yiheng Technology Co., Ltd., shanghai China) for 60 min, after which the amplification results were visually inspected via colorimetric changes, with violet indicating a negative result and sky-blue a positive result.

With regard to sensitivity tests, each HPV DNA plasmid solution was serially diluted to 10^8^, 10^7^ and 10^6^ copies/μl. The number of viral DNA copies was obtained using the following formula:

$$ Number\ of\ virus\ copies=\frac{6.02\times {10}^{23}\times DNA\  concentration}{DNA\  length\times 660} $$

Six of the eight wells on each microfluidic chip were pre-treated with different HPV primer sets while the remaining two wells remained empty and served as negative controls. The primer-free LAMP reaction solutions containing 1 μl of HPV-L1 plasmids at different concentrations were separately injected into respective chips, followed by amplification using a 60 °C water bath for 60 min. The results were then visually inspected as described previously.

### DNA extraction from skin scrapes of patients

The genomic DNA of skin scrapes were extracted using an AxyPrep Multisource Genomic DNA Miniprep Kit (Axygen Scientific Inc., USA) according to the protocol provided by the manufacturer. In brief, skin scurf from the wart lesion of the patients was harvested with use of a sterile scalpel blade and deposited into a 1.5 ml EP tubes. Scurf samples were ground with PBS buffer dissolved with RNase A using a pre-cooled mortar and pestle. Tissue homogenate was then collected and lysed at 56 °C for 10 min. Cellular debris pellets were removed by centrifugation, the DNAs from the supernatant were isolated by centrifugation, the DNAs were eluted with Eluent, and finally quantified with use of a UV-Vis Spectrophotometer Q500 instrument (Quawell).

### PCR amplifications and evaluation of LAMP reactions

For evaluation of HPV detection with LAMP microfluidic chips, reaction buffer containing 3 ng of each clinical DNA sample was loaded onto the low-risk type HPV LAMP microfluidic chip. The reaction buffer and amplification conditions were set as above. Detection results were confirmed through direct visual color inspection described previously. These same DNA samples were amplified via conventional PCR reactions with the use of a pair of HPV universal primers (FAP6085/64) [32], the sequence of which was:

- FAP6085: 5′-CCWGATCCHAATMRRTTTGC-3′
- FAP64: 5′-CCWATATCWVHCATITCICCATC-3′.

In order to determine the lower detection limit of the PCR, six HPV types (HPV1, HPV2, HPV3, HPV4, HPV27, and HPV57) were selected for PCR detection using universal HPV primers. 10^8^ copies/μl, 10^7^ copies/μl and 10^6^ copies/μl HPV plasmid concentrations were selected for three PCR tests. The PCR reaction system included 2.5 μl of 10 × pfu Buffer, 0.5 μl of dNTP (0.2 μM), 2 μl of pfu DNA polymerase (2.5 U), 8 μl of each primer (20 μM), 2 ng of DNA and nuclease-free water to obtain a final volume of 25 μl. The PCR reaction conditions included, pre-heating of samples at 95 °C for 5 min, followed by 60 cycles of 50 s at 94 °C, 50 s at 49 °C and 50 s at 72 °C. When performing PCR detection of clinical samples, the PCR products were then subjected to a 1% agarose gel electrophoresis assay, after which the target DNA fragments were recycled with use of the Omega gel extraction kit (Omega Inc., USA) following instructions provided and sequenced in cooperation with The Beijing Genomics Institute. Genotypes of HPVs were identified by comparing the acquired sequences against the NCBI nucleotide database. The specificity of LAMP microfluidic reactions was then evaluated by comparing results from both LAMP reactions and sequencing of PCR products.

### Statistical analyses

The sensitivity and specificity of the LAMP microfluidic chip detection system were evaluated with use of the statistical software named “Diagnostic test evaluation calculator”. Consistency test (kappa) was used to compare results from the two detection methods using SPSS software.

## Results

### Evaluation of the microfluidic LAMP reaction HPV detection system

**Fig. 1:**
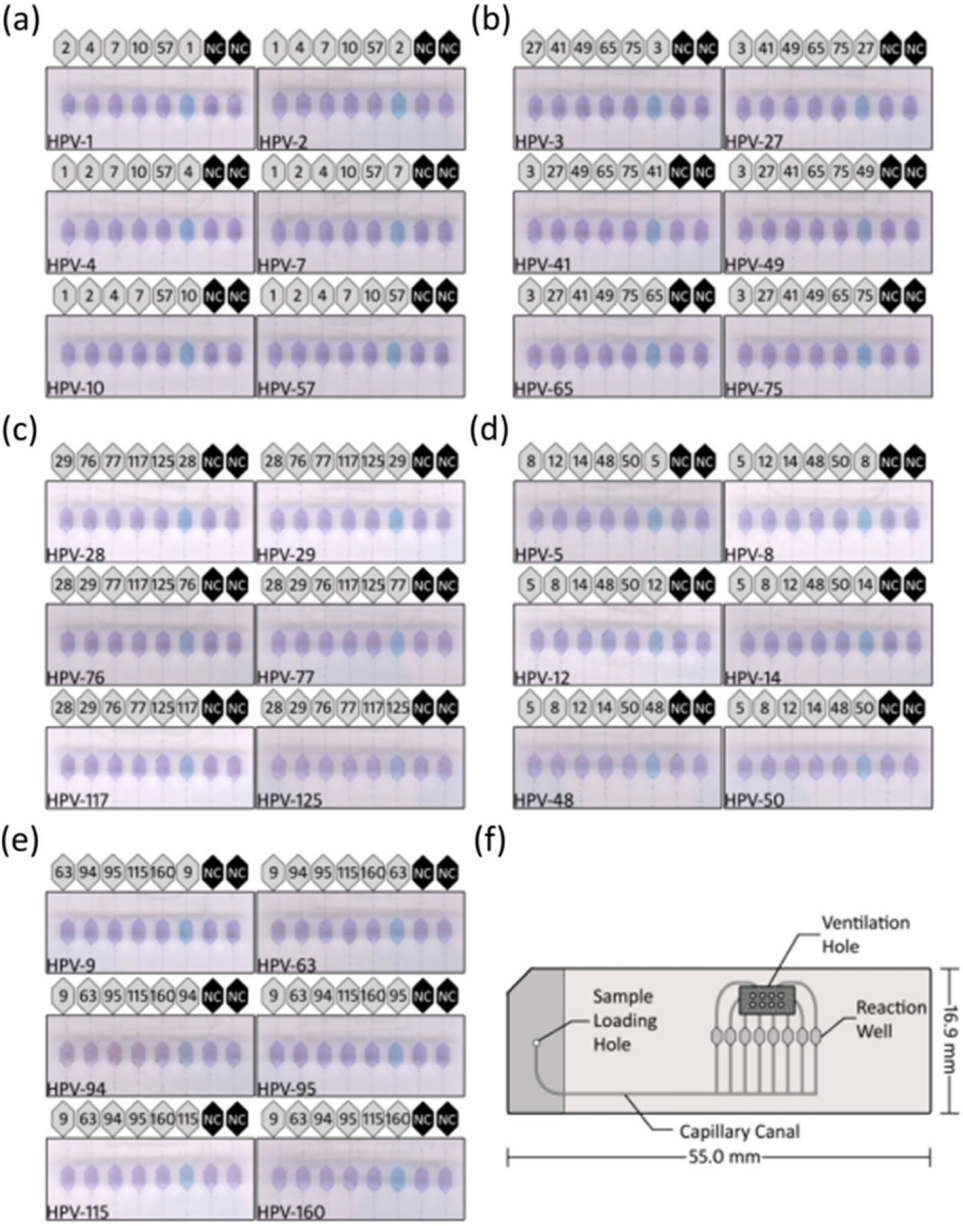
Specificity tests within groups. In one chip, LAMP reactions were performed between the six HPV type primers and the HPV type plasmid in the sixth well. When the first 5 wells and two NC wells are negative (violet) and the 6th well is positive (sky blue), it means that the HPV type primers of the first 5 wells have good specificity for the HPV type plasmid of the 6th well. **a** Specificity detection of group 1 in the low risk type group. The specificity of the six HPV type primers (HPV1, 2, 4, 7, 10 and 57) in the group was verified. **b** Specificity detection of group 2 in the low risk type group. The specificity of the six HPV type primers (HPV3, 27, 41, 49, 65 and 75) in the group was verified. **c** Specificity detection of group 3 in the low risk type group. The specificity of the six HPV type primers (HPV28, 29, 76, 77, 117 and 125) in the group was verified. **d** Specificity detection of high risk type group. The specificity of the six HPV type primers (HPV5, 8, 12, 14, 48 and 50) in the group was verified. **e** Specificity detection of rare type group. . The specificity of the six HPV type primers (HPV9, 63, 94, 95, 115 and 160) in the group was verified. **f** Microfluidic chip diagram

**Fig. 2:**
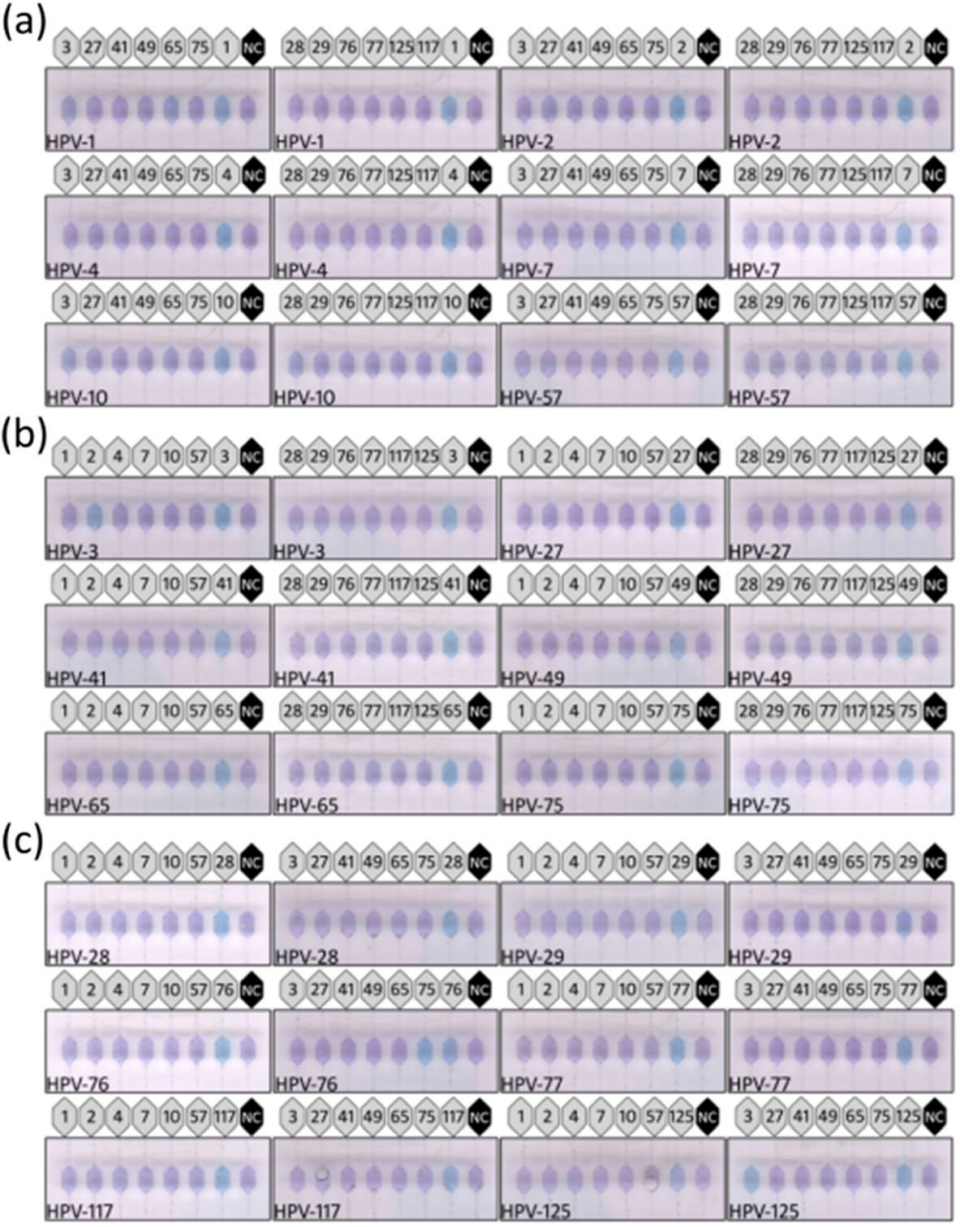
Specificity tests among three low-risk type HPV groups. In one chip, LAMP reactions were performed between the 7 HPV type primers and the HPV type plasmid of the 7th well. When the first 6 wells and one NC well are negative (violet) and the 7th well is positive (sky blue), it means that the HPV type primers of the first 6 wells are specific to the HPV type plasmid of the 7th well. When a positive reaction occurs in the first 6 wells, it means that the HPV type primer in this well has a non-specific cross-reaction with the HPV type plasmid in the 7th well. **a** Specificity detection of group 1 against group 2 and 3. Using group 2 and 3 HPV plasmids to detect the specificity of group 1 HPV primers. **b** Specificity detection of group 2 against group 1 and 3. Using group 1 and 3 HPV plasmids to detect the specificity of group 2 HPV primers. **c** Specificity detection of group 3 against group 1 and 2. Using group 1 and 2 HPV plasmids to detect the specificity of group 3 HPV primers

**Fig. 3:**
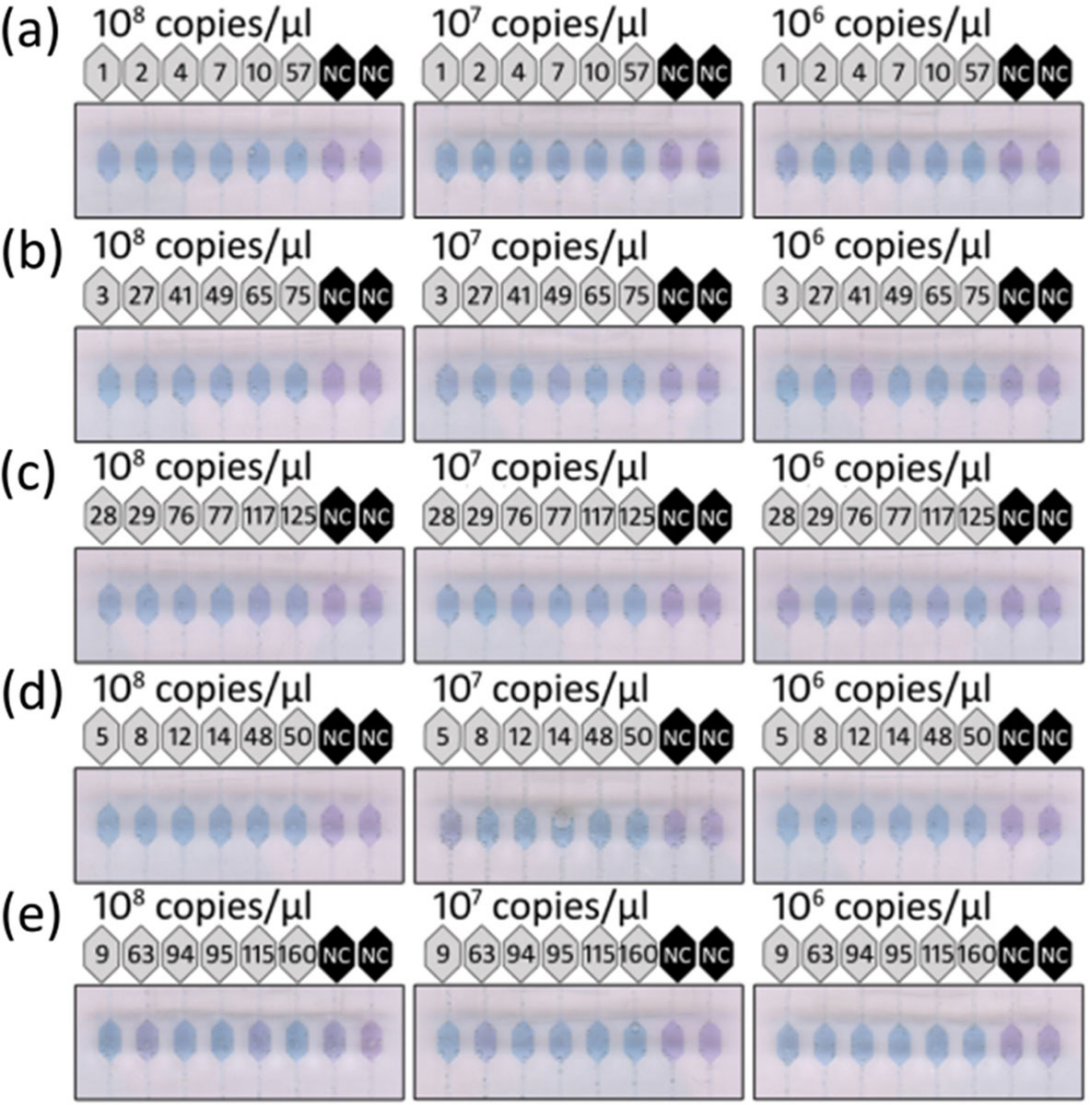
30 Kinds of Cutaneous HPV sensitivity tests. In one chip, LAMP reactions were performed between 6 HPV type primers and a mixture of these 6 HPV type plasmids at a certain concentration. When the first 6 wells are positive (sky blue) and the two NC wells are negative (violet), it means that these 6 HPV primers can produce a positive reaction with this concentration of HPV type plasmid. Positive amplifications occurred at a plasmid concentration of 10^7^ copies/μl for all primers tested, but not all primers showed positive reactions at 10^6^ copies/μl. The lowest concentration of HPV DNA that the detection system can detect was set to 10^7^ copies/μl. **a** Sensitivity detection of group 1 in the low risk type group. The HPV plasmid concentrations of 10^8^, 10^7^ and 10^6^ copies/μl were selected for sensitivity detection of HPV primers in group 1of low risk type group. **b** Sensitivity detection of group 2 in the low risk type group. The HPV plasmid concentrations of 10^8^, 10^7^ and 10^6^ copies/μl were selected for sensitivity detection of HPV primers in group 2 of low risk type group. **c** Sensitivity detection of group 3 in the low risk type group. The HPV plasmid concentrations of 10^8^, 10^7^ and 10^6^ copies/μl were selected for sensitivity detection of HPV primers in group 3 of low risk type group. **d** Sensitivity detection of high risk type group. The HPV plasmid concentrations of 10^8^, 10^7^ and 10^6^ copies/μl were selected for sensitivity detection of HPV primers in high risk type group. **e** Sensitivity detection of rare type group. The HPV plasmid concentrations of 10^8^, 10^7^ and 10^6^ copies/μl were selected for sensitivity detection of HPV primers in rare type group

Given the consideration of time and expenses costs, the 30 cutaneous HPVs were divided into three major groups and five minor groups for testing according to the type of disease and their common degree. There are 18 low-risk HPVs in 3 minor groups (major group 1), 6 high-risk HPVs in minor group 4 (major group 2) and 6 rare HPVs in minor group 5 (major group 3). No cross reactivity was observed within each of the five minor groups during specificity tests (Fig. 1). However, between the three low-risk type groups, the HPV3 plasmid was amplified by HPV2 primers, HPV125 plasmid by HPV3 primers and HPV76 plasmid by HPV75 primers (Fig. 2). No cross reactivity was observed among the other HPV types in the low-risk type groups. For sensitivity tests, all 30 HPV-L1 plasmids were serially diluted as mentioned above and amplified by their specific LAMP primers. Results revealed that positive amplifications occurred at a plasmid concentration of 10^7^copies/μl for all primers tested (Fig. 3). Therefore, maximal sensitivity of the LAMP detection system was obtained with 10^7^ copies of HPV-DNA per microliter.

### Detection of clinical samples using microfluidic chips

**Fig. 4:**
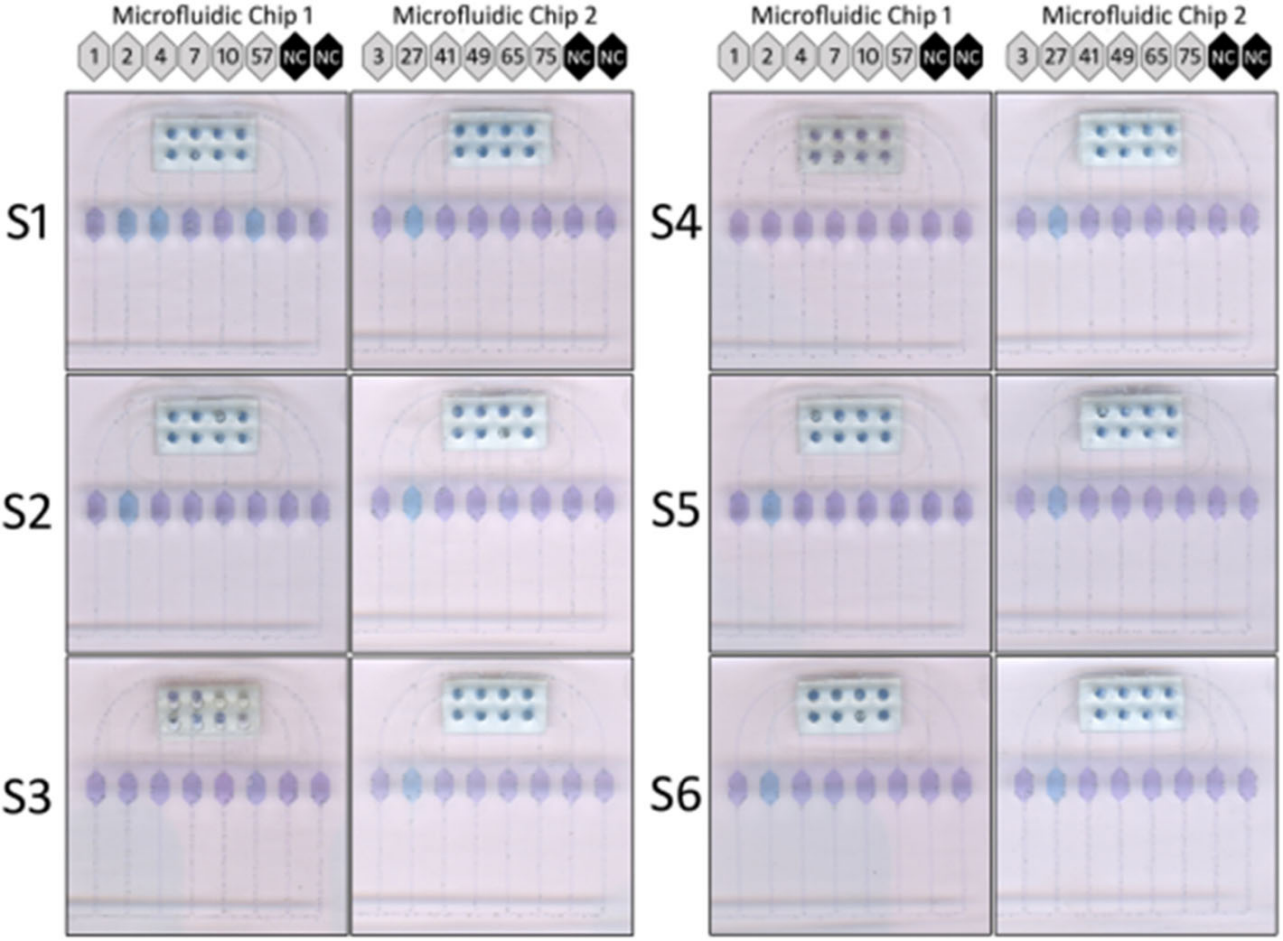
Test results of the first six clinical samples. In one chip, LAMP reaction was performed between 6 HPV type primers and clinical sample DNA. When one or more positive reactions (sky blue) appear in the first 6 wells and the two NC wells were negative (violet), it means that the corresponding HPV type infection was detected in this clinical sample. S1–6 indicates the first 6 samples tested, and each sample tested the HPV type in group 1 and group 2 of low-risk HPV. Positive wells in the two chips of each sample indicate detection of the corresponding HPV type

In order to assess the efficacy of the new HPV detection system, we used the chips established for low-risk type HPV sub-groups 1 and 2 for the tests on clinical samples. DNA samples from 85 patients were amplified individually and detected using either microfluidic LAMP reactions followed by visual inspection or by conventional PCR followed by sequencing. Detailed test results are presented in Supplementary Table 2. The microfluidic LAMP detection results for the first 6 samples are shown in Fig. 4. With the microfluidic LAMP detection system, HPV sample detection rates was 100% as compared to 72.9% for the conventional PCR and sequencing method. The multiple infection ratio detected by microfluidic LAMP was 64.7%, whereas no multiple infections were detected with use of conventional PCR and sequencing (Table 1). The sensitivity of LAMP detections for HPV27, HPV2, HPV1, HPV57 and HPV3 was 100% as compared with that of PCR sequencing, while the specificity was 34.55, 45.12, 95.83, 98.59 and 97.62%, respectively (Table 2). When comparing these two detection methods, a fair agreement was found for HPV27, a slight agreement for HPV2, an almost perfect agreement for HPV1 and HPV57 and a moderate agreement for HPV3. We also tested samples with a single HPV infection, the sensitivity of LAMP detections for HPV27, HPV2, HPV1 and HPV57 was 100%, whereas the specificity was 61.11, 89.66, 100, 100 and 96.67%, respectively, when compared with that obtained using PCR sequencing. The agreements between the two methods were found to be moderate for HPV27, fair for HPV2 and perfect for both HPV1 and HPV57 (Supplementary Table 3).

**Table 1:** Clinical sample HPV infection rate detected by LAMP and PCR & Sequencing: This table lists the single infection rate and multiple infection rate when using the PCR & Sequencing method and the microfluidic LAMP method to detect clinical samples, respectively

	Single infection	Multiple infection	Double infection	Triple infection	Quartet infection	HPV positive	HPV negative	Total
	n(n/N)	n(n/N)	n(n/N)	n(n/N)	n(n/N)	n(n/N)	n(n/N)	N
LAMP	30 (35.3%)	55 (64.7%)	42 (49.4%)	12 (14.1%)	1 (1.2%)	85 (100%)	0 (0)	85
PCR	62 (72.9%)	0 (0)	0 (0)	0 (0)	0 (0)	62 (72.9%)	23 (27.1%)	85

LAMP represents the new HPV detection system based on LAMP and microfluidic chip. PCR represents DNA sequencing based on PCR methods

*Abbreviations*: *HPV* human papillomaviruses, *LAMP* Loop-mediated isothermal amplification, *PCR* Polymerase Chain Reaction

**Table 2:** Comparison of test results of both LAMP and PCR & Sequencing in clinical sample detection: The PCR & Sequencing method was used as the standard, the sensitivity and specificity of the microfluidic LAMP method used to detect the clinical samples were analyzed

LAMP	PCR		Total	Sensitive (%)	Specificity (%)	Kappa
	Positive	Negative		95% CI	95% CI	
HPV27						
Positive	30	36	66	100 (88.43–100)	34.55 (22.24–48.58)	0.271 (*P* < 0.001)
Negative	0	19	19			
Total	30	55	85			
HPV2						
Positive	3	45	48	100 (29.24–100)	45.12 (34.1–56.51)	0.055 (*P* > 0.05)
Negative	0	37	37			
Total	3	82	85			
HPV1						
Positive	13	3	16	100 (75.29–100)	95.83 (88.3–99.13)	0.876 (*P* < 0.001)
Negative	0	69	69			
Total	13	72	85			
HPV57						
Positive	14	1	15	100 (76.84–100)	98.59 (92.40–99.96)	0.958 (*P* < 0.001)
Negative	0	70	70			
Total	14	71	85			
HPV3						
Positive	1	2	3	100 (2.5–100)	97.62 (91.66–99.71)	0.491 (*P* < 0.001)
Negative	0	82	82			
Total	1	84	85			
HPV4						
Positive	0	3	3	–	96.47 (90.03–99.27)	–
Negative	0	82	82			
Total	0	85	85			
HPV7						
Positive	0	1	1	–	98.82 (93.62–99.97)	–
Negative	0	84	84			
Total	0	85	85			
HPV75						
Positive	0	1	1	–	98.82 (93.62–99.97)	–
Negative	0	84	84			
Total	0	85	85			

LAMP represents the new HPV detection system based on LAMP and microfluidic chip. PCR represents DNA sequencing based on PCR method

*Abbreviations*: *HPV* human papillomaviruses, *LAMP* Loop-mediated isothermal amplification, *PCR* Polymerase Chain Reaction, *CI* confidence interval

## Discussion

Cutaneous HPVs are classified into low-risk or high-risk type according to the different malignancy levels of the diseases they cause. In this study, we categorized the 30 cutaneous HPVs into three groups, namely low-risk, high-risk and rare-type groups. All clinical samples were initially subjected to both low and high-risk groups, and to the rare-type group only when the former two were negative. The newly established LAMP HPV detection system contained 5 reaction groups which were set according to the genotypes, with each consisted of 6 different HPVs. The low-risk group consisted of 3 minor groups including 18 low-risk HPV types; the high-risk group and the rare group each had 1 minor group including 6 HPV types. There were no cross-reactions between the 6 HPV type primers in each minor group. However, three specific cross-reactions occurred between the 3 low-risk minor groups namely HPV2 with HPV3, HPV3 with HPV125 and HPV75 with HPV76. DNA alignments of these HPVs indicated that the L1 gene sequence homology was 66.1% for HPV2 and HPV3, 85.4% for HPV3 and HPV125 and 88.2% for HPV75 and HPV76. Given that any HPV that possesses 10% of L1 gene heterogeneity was recognized as a new species, therefore we believe the cross-reactions observed within our LAMP detections may very likely due to a high DNA sequence homology.

The lower detection limit of the LAMP HPV detection system was around 10^7^ copies of HPV per microliter. LAMP detection technology is more sensitive than other nucleic acid detection technologies, which also leads to an increase in the false positive rate of LAMP. The more virus types and the higher the virus homology is, the higher the change of false positive reactions. In order to reduce the false positive rate of 30 HPV type detections, this study reduced the sensitivity when designing primers. Previous studies have shown that HPV has a high viral load in skin warts, with a concentration of 66,706 genome copies per single cell [33]. Therefore, the sensitivity of this study can fully meet the detection needs of wart samples, and it should be further enhanced like subtle adjustments in the design of the primers for future work in this area.

There were three specific cross-reactions using HPV containing plasmid, while there was no cross-reactivity in the clinical sample detection. This may be associated with lower concentrations of HPV DNA in clinical samples, which increases the specificity of the LAMP method. The main reason for the low specificity and limited agreements primarily attributes to the inability of the PCR sequencing method to detect and distinguish multiple HPVs infections with one run of the assay. This possible explanation receives support as based upon results obtained with our newly established LAMP detection system in the clinical samples (reaching 64.7% of all samples tested). When testing samples with a single HPV infection, the specificity of the LAMP detection and agreement of two methods was improved, suggesting that the low specificity of LAMP detection and limited agreements were mainly due to the relatively low sensitivity of PCR sequencing, which then results in failures in detecting HPVs at lower titers. The gold standard detection method used for HPV detection, PCR sequencing, uses the cutaneous universal primer FAP6085/64. FAP primers are the most common universal primers that can amplify multiple HPV types. FAP6085/64 can detect more than 300 PV types and more than 100 unknown HPV types in healthy skin [32]. However, we ran a lower detection limit PCR experiment and the results showed that the sensitivity of PCR reaction using HPV universal primers was 10^8^ viral DNA copies/μl (Supplementary Figure 1). The result indicated the sensitivity of PCR detection for viruses is much lower than that of LAMP, which makes it difficult to precisely judge the sensitivity and specificity of the new HPV detection system. Therefore, a more competent and robust reference detection method will be required in order to further evaluate the LAMP detection system. Notably, only benign skin warts were collected in this study as test samples to validate the newly established LAMP detection system, samples from other skin diseases and healthy skin were not collected, which results in an insufficient case detection rate (CDR) with high-risk HPVs. Therefore, in order to achieve a more comprehensive evaluation of the LAMP HPVs detection system, more clinical samples from non-melanoma skin cancers or other high-risk HPV related cutaneous lesions will be required in future investigations.

This newly established LAMP detection system demonstrates some notable advantages over the conventional PCR sequencing method. For example, LAMP reactions identified multiple cutaneous HPV infections in clinical samples and possessed greater degrees of sensitivity with good specificity. With use of the LAMP detection system, we found that HPV27, HPV2, HPV1, HPV57, HPV3, HPV4, HPV7 and HPV75 were the most common types of HPV infections revealed within the Shenyang area of China. The most common multiple infection was the combination of HPV2 and HPV27. The HPV sample detection rate with the LAMP detection system was 100%, as compared with 73% from PCR sequencing, demonstrating that the new LAMP detection method was more effective in detecting cutaneous HPVs within the clinical setting. Interestingly, of the 85 clinical samples tested with use of PCR sequencing, one case (sample 72) was infected with HPV-11, but there is no HPV11 in the LAMP method and the LAMP method only detected HPV2 and 27 in this sample. This specific case was mucosal type, suggesting the possibility that low-risk types of mucosal HPVs could also occur in benign skin warts. At present, the detection of cutaneous HPV basically depends on the PCR method, and the number of cutaneous HPV types detected is small. The results of other studies are consistent with the HPV types of common skin warts detected in our study [34, 35]. The multiple infection rate of the detection system in this study was high at 64.7%. Double infection was the most common, and the maximum number of HPV types infected at the same time was 4. The multiple infection rate of skin warts in related studies were lower than that of our study, which may be related to the limitations of the PCR method.

These LAMP reactions are very easy to implement, due to the simple amplification conditions and the intuitive visual inspection of the results. Combined with microfluidic chip technology, the cutaneous HPVs detection system can be readily operated with minimal equipment and by personnel with limited expertise. The HPVs detection system in this study can be applied clinically in two areas. One is to achieve a more precise medical treatment of skin warts. There are various treatments for skin warts in the clinic, and even for patients with the same kind of skin warts, the treatment effects differs among individuals. In the future, we can analyze the treatment effectiveness with the HPVs detection results to find a better treatment method for a certain HPV type infection. The second is to assist the diagnosis of malignant skin tumors. The diagnosis of malignant skin tumors has relied on skin pathological tests. With HPV type detection added to the detection method of malignant skin tumors, it can help the identification and diagnosis of malignant skin tumors. In this study, the skin warts samples were collected for low risk type HPV (sub-groups 1 and 2) detection. The remaining 18 HPV types (high risk and rare type HPVs) were only tested for HPV containing plasmids, and additional clinical samples such as basal cell carcinoma will be required for further test and validation of clinical implementations. The 85 clinical evaluated samples in our current report provided robust evidence serving as a foundation for implementation of this technique. There have been relevant studies on the use of LAMP for cervical cancer screening. It aims at the typing of mucosal high-risk HPV and has a good clinical application possibility [36]. It also confirms the significance and feasibility of this study. We believe that the application of this cutaneous HPVs detection system will provide increased information to enable a more effective clinical judgement regarding cutaneous malignancies and epidemiological evidence for vaccine development, contribute to etiologic diagnosis, and be able to prescribe more precise and specialized therapies.

## Conclusion

In this study, we established a rapid detection system for cutaneous Human Papillomaviruses (HPVs) using colorimetric loop-mediated isothermal amplification (LAMP) in combination with microfluidic technology. Such a system provides a more efficient, rapid and cost effective means for use in detecting cutaneous HPVs.

## Supplementary information

**Additional file 1: Supplementary Figure 1.** Sensitivity test of PCR. PCR reactions were performed on 6 kinds of HPV type plasmids (HPV1, HPV2, HPV3, HPV4, HPV27 and HPV 57) with universal HPV primers. (A) 10^8^ copies/μl, (B) 10^7^ copies/μl and (C) 10^6^ copies/μl plasmid concentrations were selected for three PCR tests.

**Additional file 2: Supplementary Table 1.** HPV primer sequences: This table lists the LAMP primer sequences of 30 HPVs in this study. Each HPV primer consists of four primer sequences, which are F3, B3, FIP, and BIP. **Supplementary Table 2.** HPV detection test results of clinical samples: This table lists HPV detection test results using the microfluidic LAMP method and PCR method for each clinical sample. **Supplementary Table 3.** Comparison of test results of both LAMP and PCR & Sequencing in clinical samples infected with only one type of HPV: The PCR & Sequencing method was used as the standard, the sensitivity and specificity of the microfluidic LAMP method used to detect the clinical samples were analyzed. This table analyzes only clinical samples infected with one type of HPV.

## Data Availability

All data generated or analyzed during this study are included in this published article.

## Abbreviations

HPV: Human papillomavirus
AKs: Actinic keratosis
NMSCs: Non-melanoma skin cancers
LAMP: Loop-mediated isothermal amplification
HNB: Hydroxynaphthol blue
CDR: Case detection rate
