# Supplementary material for: Genotyping of 30 kinds of cutaneous human papillomaviruses by a multiplex microfluidic loop-mediated isothermal amplification and visual detection method
Source: Virol J. 2020 Jul 9;17:99. doi: 10.1186/s12985-020-01373-3 (PMC7345449; doi:10.1186/s12985-020-01373-3)
Supplement: Supplementary file 1 — Additional file 1: Supplementary Figure 1. Sensitivity test of PCR. PCR reactions were performed on 6 kinds of HPV type plasmids (HPV1, HPV2, HPV3, HPV4, HPV27 and HPV 57) with universal HPV primers. (A) 108 copies/μl, (B) 107 copies/μl and (C) 106 copies/μl plasmid concentrations were selected for three PCR tests. [file 12985_2020_1373_MOESM1_ESM.docx]

**Supplementary legends**

**Supplementary figure 1. Sensitivity test of PCR.** PCR reactions were performed on 6 kinds of HPV type plasmids (HPV1, HPV2, HPV3, HPV4, HPV27 and HPV 57) with universal HPV primers. (A) 10^8^ copies/μl, (B) 10^7^ copies/μl and (C) 10^6^ copies/μl plasmid concentrations were selected for three PCR tests.

**Supplementary figure 1. Sensitivity test of PCR.**

**
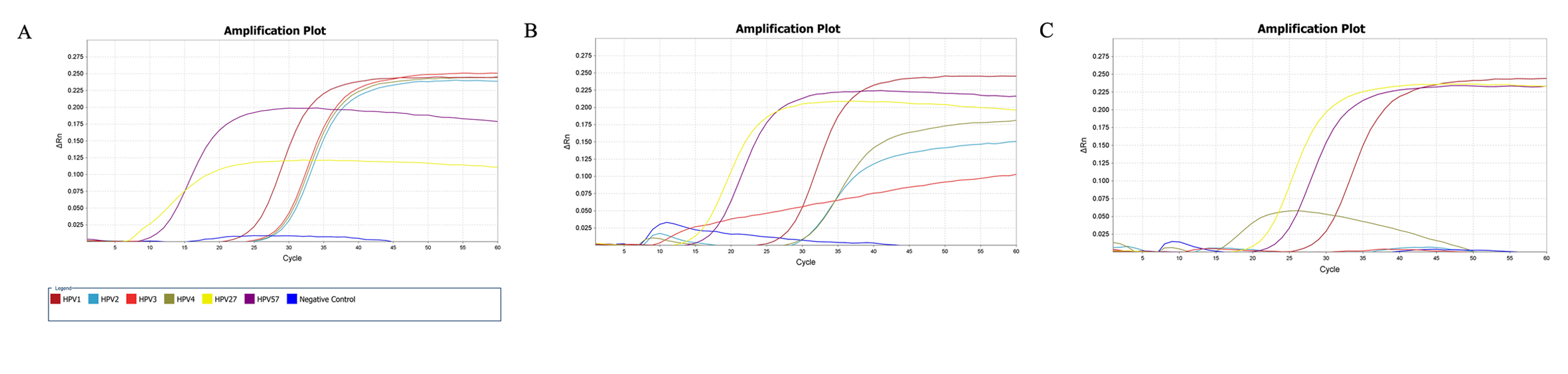
**
