# Supplementary material for: Genotyping of 30 kinds of cutaneous human papillomaviruses by a multiplex microfluidic loop-mediated isothermal amplification and visual detection method
Source: Virol J. 2020 Jul 9;17:99. doi: 10.1186/s12985-020-01373-3 (PMC7345449; doi:10.1186/s12985-020-01373-3)
Supplement: Supplementary file 2 — Additional file 2: Supplementary Table 1. HPV primer sequences: This table lists the LAMP primer sequences of 30 HPVs in this study. Each HPV primer consists of four primer sequences, which are F3, B3, FIP, and BIP. Supplementary Table 2. HPV detection test results of clinical samples: This table lists HPV detection test results using the microfluidic LAMP method and PCR method for each clinical sample. Supplementary Table 3. Comparison of test results of both LAMP and PCR & Sequencing in clinical samples infected with only one type of HPV: The PCR & Sequencing method was used as the standard, the sensitivity and specificity of the microfluidic LAMP method used to detect the clinical samples were analyzed. This table analyzes only clinical samples infected with one type of HPV. [file 12985_2020_1373_MOESM2_ESM.docx]

**Supplementary Table legends**

**Supplementary table 1. HPV primer sequences**: This table lists the LAMP primer sequences of 30 HPVs in this study. Each HPV primer consists of four primer sequences, which are F3, B3, FIP, and BIP.

**Supplementary table 2. HPV detection test results of clinical samples**: This table lists HPV detection test results using the microfluidic LAMP method and PCR method for each clinical sample.

**Supplementary table 3. Comparison of test results of both LAMP and PCR & Sequencing in clinical samples infected with only one type of HPV**: The PCR & Sequencing method was used as the standard, the sensitivity and specificity of the microfluidic LAMP method used to detect the clinical samples were analyzed. This table analyzes only clinical samples infected with one type of HPV.

**Supplementary Table 1**. **HPV primer sequences**

| NO. | HPV type | Primer | Gene |
| --- | --- | --- | --- |
| 1 | HPV1 | F3 | GCTGTACTCCTGCTTCAG |
|  |  | B3 | TGCATGTTGCTTGAACAAC |
|  |  | FIP | GACTCTATCATTTGCACCCTGGGACACTGGACAAGTAGTCGT |
|  |  | BIP | GATGGTGACATGATGGATATTGGTGGGACATCAGACTTGTCTTG |
| 2 | HPV2 | F3 | CTGCCAGTTTACAGGATACC |
|  |  | B3 | AGACACGGTAGGCATAGC |
|  |  | FIP | TCGGTAGGGGTCTTAGGAGGGATATTTGCAGTCCCAGGC |
|  |  | BIP | GACCTTTTGGGATGTGGATCTCAGCGCTGCAGCAAAAACTTG |
| 3 | HPV3 | F3 | TGGGTTGTACCCCACCTATG |
|  |  | B3 | GCAGGTGGTCTGGCAAAT |
|  |  | FIP | GGAGGACAATCACCCGGTGACGGGAGCATTGGGGCAAAG |
|  |  | BIP | GGCGATATGGTGGACACAGGTGGCACGTCTGACTTATTGGA |
| 4 | HPV4 | F3 | CCTGCAATAGGTGAACATTG |
|  |  | B3 | GGCCATTTACAAACTGTGG |
|  |  | FIP | AGCTCTATTGGTGGGCAATCTCGGATAAAGCTGAACCTTGTC |
|  |  | BIP | ATGTGTGACATTGGATTTGGGGGCACTAGATTTATCAGCCTG |
| 5 | HPV5 | F3 | GATCCAAATGTTTATTGTAGGATG |
|  |  | B3 | ATTGACGATGTCTAAACTGAC |
|  |  | FIP | GGACAAAGGCCAGTTTGCTGGGAGAGCATTGGGATAAAGC |
|  |  | BIP | ATACAAGATGGTGATATGGCAGACCTGATCTACTATCTTGAAGTGC |
| 6 | HPV7 | F3 | GAGTGTTTAGAGTACGCTTG |
|  |  | B3 | CAGACGAGTTTTCCACATCT |
|  |  | FIP | CAAGGCGTTGGGTTTCAGAATTCCCGACCCTAATAAATTTGG |
|  |  | BIP | ATGGGCCTGTGTTGGTGTTGGTATGGATGACCACTAATGC |
| 7 | HPV8 | F3 | TGTTTTAGCACAAATCAATGC |
|  |  | B3 | CATTCCAGAAGTTAAACTTTGC |
|  |  | FIP | TTGGAGTATCAGGGGTAGGTACCATGAATTCATCACTACTTGAGG |
|  |  | BIP | TGATTCTCTTGCCACACGTTGATATGGATCAGGCTTTTCCTTAG |
| 8 | HPV9 | F3 | ACCGTTTGCTAACAGTGG |
|  |  | B3 | GCCTATTTCAATACCTCTACAG |
|  |  | FIP | ACTGATTACCAGACACTTTAGGGACTCCATATTATGATGTCCGCTC |
|  |  | BIP | AGCTTACCTGATCCAAATAGGTTTGGCCCAAACTAGACGTTCC |
| 9 | HPV10 | F3 | CATATTAAAGAGCAACGGTGG |
|  |  | B3 | TCAGAAGGAACACACAAGC |
|  |  | FIP | AGCCTCAGACGTTACCATGGGACGTTGGTAGTGCTGTG |
|  |  | BIP | CCAGGGGCACAACAATGGTAACTGCGAGTCGTGTCTAC |
| 10 | HPV12 | F3 | CTCAAATAACTATGCCACAGG |
|  |  | B3 | GTCACCATCTTCAATGAAAGTG |
|  |  | FIP | GTACAGCCCACTATAAACATTTGGACAGTAAGGATGATAGACAGAAC |
|  |  | BIP | CCTTGTGTGGGAGAACACTGGCTCTATGGGAGGGCAAAC |
| 11 | HPV14 | F3 | AGGTATAGAAATAGGCAGAGG |
|  |  | B3 | TTCTACACATGGCAAGGC |
|  |  | FIP | GAGTTGGGATTTTCTGTATCACCAAACAACCTTTAGGTGTAGGTAG |
|  |  | BIP | TGTGTCATTTGATCCTAAGCAACTGAATGTTCCCCCATGCAAG |
| 12 | HPV27 | F3 | CCAATAGGTCTGATGTTCCTT |
|  |  | B3 | GGTCCGAGATAGTGGTACT |
|  |  | FIP | AAGGCTCTGCAGCCATTTTCTGGATATTTGTACAAACGTCTG |
|  |  | BIP | TTCGCTGCGTAGAGAACAGATCTCATCTGGGATTGTGTCAC |
| 13 | HPV28 | F3 | CACAACAGGGAGATTGCC |
|  |  | B3 | AACATGCTGTCGCCATAC |
|  |  | FIP | CATTGCACCAAAGCCAGTGTGAGCTTATAACTGCCCCTAT |
|  |  | BIP | TACAGGCCAATAAATCGGACGTGCCATGCCTAAATAATCAGG |
| 14 | HPV29 | F3 | ACAGAGTCTCAACCGTTG |
|  |  | B3 | CGTGTCTTCCAAGCTAGTG |
|  |  | FIP | CTGCAAATCATATTCCTCCCCACCACTTATGATGCTACCAAGA |
|  |  | BIP | TGACACCTGAAATTATGGCTTACCGGTGGCAATGTCAATCCAA |
| 15 | HPV41 | F3 | TACTTTCCTCCATGCTGC |
|  |  | B3 | AACCTCAATCCCACGAAT |
|  |  | FIP | ACCTCTTTGCCATCCGCATTCCGTTTGCTTACTGTTGG |
|  |  | BIP | TTCCGTGTCCGTTTCCCAAACGCTCCTTGTCAGGGTTA |
| 16 | HPV48 | F3 | GAGACTCTGTCTTCTTTTTTGG |
|  |  | B3 | CGTCTAAGCCAATAAGGCC |
|  |  | FIP | TGGTATAGGCTCCCCCATTTGACGGGAACAATGTTATGCT |
|  |  | BIP | CTGCCGATCAAAACAACAGATCTCTAGTATTCAAGGATCCACTTGG |
| 17 | HPV49 | F3 | CCTGCAGCAAGTCAACAG |
|  |  | B3 | GCCATCCGTACTTACACTA |
|  |  | FIP | GGTCTATTGAATAGCTGCGCATCAGGCAGCTCCATCTATTTCC |
|  |  | BIP | TTGGTTACAAAGAGCACAGGGTGGTATTATCAGCCACTGTT |
| 18 | HPV50 | F3 | GGATGCTGATATATTAGCTCATCT |
|  |  | B3 | TTTCTGTAAGGTTGACATTCC |
|  |  | FIP | CGCCTATACCACTGGGAGCTCCAGAATTTTGGAAAATTGGC |
|  |  | BIP | TCTGATGCTACAAAATGTCCTGCTTCTTTATAAGGGTCTACTACTTCG |
| 19 | HPV57 | F3 | CCGGATGAGCTATATGTCAAG |
|  |  | B3 | ACAAAGAGACATTTGTGCTG |
|  |  | FIP | CCATAGAGCCACTGGGAGTGAGTTCTACCGTCCAGACC |
|  |  | BIP | AACAAGCCTTACTGGCTGCGTTAGGAAGATCCGATTGCC |
| 20 | HPV63 | F3 | TTCCTACCCAACCGATCA |
|  |  | B3 | TTATCTCCAAAGGCAAATCG |
|  |  | FIP | ATCACTGGTAGCGTGATAGAAGATGCCAAGATTCTAAGCAGCGA |
|  |  | BIP | ATGAGGTTACCCGTGCAAATGATAAGGAAATCTAACACGAAAGACTC |
| 21 | HPV65 | F3 | CCATTGGATGTAGTTGCTAC |
|  |  | B3 | ATCCTGACCTTCTTGAGC |
|  |  | FIP | CTCGCCTACCATAAAAGAATAAGCTAATGTGCAAATGGCCAGAT |
|  |  | BIP | TATGCCAGGCACTTTTTTGTTAGAGCCAGTAATCAGTTTTAACCTCG |
| 22 | HPV75 | F3 | CCTTAAAATGGCCAATGACA |
|  |  | B3 | CGTGGGAACATAAATAGAGTTG |
|  |  | FIP | CCTCCCCTAACAAAAAAGTGTCTAGGGACTCCTGTTTTTTTTATGCC |
|  |  | BIP | GATGCAATTCCTAATGCTGCAGTCCCAAAGTGTTTTGAGCC |
| 23 | HPV76 | F3 | TCCTTACTGTAGGCCACC |
|  |  | B3 | ACCTCTACAGGCCCAAAC |
|  |  | FIP | TGCTCTAAACTGATTCCCTGATACTGTTAGAGACACTGGGGATTC |
|  |  | BIP | ACTACCAGATCCCAATAGATTCGCAGTCTTTCCTTCTCAGGATT |
| 24 | HPV77 | F3 | TACTACCCCAGGAGACTG |
|  |  | B3 | AAACAGTTGTTCCCGACG |
|  |  | FIP | ACCATACCCGGTATCCACCATTCCCTTGGAGTTAATGACAT |
|  |  | BIP | GTGCCCCTTGATATTTGCCAGCATGCTATCGCCATACGG |
| 25 | HPV94 | F3 | GACTTCACTGCATTACAGTT |
|  |  | B3 | CCAACGTTTTGGTCACCA |
|  |  | FIP | CTGCTGCCATGCCCAAATAATGCCTATAGATATTTGCCAGTC |
|  |  | BIP | GACATTTTTTCAATAGAGCCAGTGCGCCGACTTCAATATTAGAGTGT |
| 26 | HPV95 | F3 | TTCTTCTTTGGCCGAAGG |
|  |  | B3 | CGGTTAAAAAGCTGAGATTCAC |
|  |  | FIP | GGCTCTGGTAACGCATCACCACAGCTTTATGCAAGACAC |
|  |  | BIP | AATTGCTGCTCAGAGTAACCAAAACCGCTAGGAGTTCCAA |
| 27 | HPV115 | F3 | ACATACAAAGGACTGACATCT |
|  |  | B3 | GTAGTATCTACCAATGCAAACC |
|  |  | FIP | GGCTCGAACCTCAAAGTATGGTTACCACGCTAATAGTGATCG |
|  |  | BIP | CAGAACCATATCAGGTGACAGTACTCAGGTAATTTAAGTCTGAAAGC |
| 28 | HPV117 | F3 | CTAGTTCTGTTGGGGACG |
|  |  | B3 | CCACCACAGTCACAAACA |
|  |  | FIP | CAGAACCATATCAGGTGACAGTACTCAGGTAATTTAAGTCTGAAAGC |
|  |  | BIP | TCTGAGGCTCAATTGTTCAATAAGCGGTTAGCCCAACAAATACCA |
| 29 | HPV125 | F3 | CCTGATTATTTGGGCATGG |
|  |  | B3 | GTGTAGGACAATATACAGCAC |
|  |  | FIP | CCAGCAACACCAGCTCTGTTTAAGCATGTTCTTTTACCTCCG |
|  |  | BIP | CACAATCCCTGACGCCTTGTTACCAAGGACATCTCGCC |
| 30 | HPV160 | F3 | TAGGCCTCAGTGGTCATC |
|  |  | B3 | CAATCACCTGACGTGGAT |
|  |  | FIP | TAGGCATGTCCGAATGTGCACTCTGTACAACAAGCTGGA |
|  |  | BIP | AACACAACTGTGCATCCTTGGTTTACATGGCGTACCCTTA |

Abbreviations: NO., number. HPV, human papillomaviruses. FIP, forward inner primer. BIP, backward inner primer.

NOTE: F3 represents forward outer primer. B3 represents backward outer primer.

**Supplementary Table 2. HPV detection test results of clinical samples**

| Clinical sample number | LAMP | PCR |
| --- | --- | --- |
| 1 | HPV2、4、27、57 | HPV57 |
| 2 | HPV2、27 | Not detected |
| 3 | HPV27 | HPV27 |
| 4 | HPV27 | Not detected |
| 5 | HPV2、27 | HPV27 |
| 6 | HPV2、27 | Not detected |
| 7 | HPV57 | HPV57 |
| 8 | HPV1、27 | HPV1 |
| 9 | HPV2、27 | Not detected |
| 10 | HPV27 | Not detected |
| 11 | HPV27 | Not detected |
| 12 | HPV27、57 | Not detected |
| 13 | HPV1、2、27 | HPV1 |
| 14 | HPV27 | HPV27 |
| 15 | HPV3、27 | Not detected |
| 16 | HPV1、27 | HPV1 |
| 17 | HPV2、27 | HPV27 |
| 18 | HPV27 | HPV27 |
| 19 | HPV1、2 | HPV1 |
| 20 | HPV2、27、57 | HPV57 |
| 21 | HPV2、57 | HPV57 |
| 22 | HPV2、27 | HPV27 |
| 23 | HPV1 | HPV1 |
| 24 | HPV4、HPV27 | HPV27 |
| 25 | HPV27 | HPV27 |
| 26 | HPV2、57 | HPV57 |
| 27 | HPV2、27 | HPV2 |
| 28 | HPV2、57 | HPV57 |
| 29 | HPV2 | Not detected |
| 30 | HPV2、27 | HPV27 |
| 31 | HPV2、27 | Not detected |
| 32 | HPV2 | Not detected |
| 33 | HPV3、27 | HPV3 |
| 34 | HPV2 | Not detected |
| 35 | HPV2、27、57 | HPV57 |
| 36 | HPV27 | Not detected |
| 37 | HPV27 | HPV27 |
| 38 | HPV27 | HPV27 |
| 39 | HPV1、27 | HPV1 |
| 40 | HPV2、27、57 | HPV57 |
| 41 | HPV27 | Not detected |
| 42 | HPV1 | HPV1 |
| 43 | HPV27 | Not detected |
| 44 | HPV1 | HPV1 |
| 45 | HPV27 | HPV27 |
| 46 | HPV1、27 | Not detected |
| 47 | HPV2、27 | Not detected |
| 48 | HPV27、HPV57 | HPV57 |
| 49 | HPV2、27、57 | HPV57 |
| 50 | HPV27 | HPV27 |
| 51 | HPV2 | HPV2 |
| 52 | HPV2、27、75 | Not detected |
| 53 | HPV2、27 | HPV27 |
| 54 | HPV1、2、27 | HPV27 |
| 55 | HPV2、27、57 | HPV57 |
| 56 | HPV27、57 | HPV57 |
| 57 | HPV2、57 | HPV57 |
| 58 | HPV2、4、27 | Not detected |
| 59 | HPV1 | HPV1 |
| 60 | HPV2、27 | HPV27 |
| 61 | HPV2、27 | HPV27 |
| 62 | HPV2、27、57 | HPV57 |
| 63 | HPV27 | HPV27 |
| 64 | HPV2、27 | HPV27 |
| 65 | HPV2、27 | HPV27 |
| 66 | HPV2、27 | HPV27 |
| 67 | HPV2、27 | HPV27 |
| 68 | HPV2、27 | HPV27 |
| 69 | HPV2、27 | HPV27 |
| 70 | HPV27 | HPV27 |
| 71 | HPV27 | HPV27 |
| 72 | HPV2、27 | HPV11 |
| 73 | HPV1、2 | HPV1 |
| 74 | HPV27 | HPV27 |
| 75 | HPV2、27 | Not detected |
| 76 | HPV2、27 | HPV27 |
| 77 | HPV27 | Not detected |
| 78 | HPV1、2、27 | HPV1 |
| 79 | HPV7 | Not detected |
| 80 | HPV1、2、27 | Not detected |
| 81 | HPV3、27 | HPV27 |
| 82 | HPV2、27 | HPV27 |
| 83 | HPV2、27 | HPV2 |
| 84 | HPV1 | HPV1 |
| 85 | HPV1、2 | HPV1 |

Abbreviations: HPV, human papillomaviruses; LAMP, Loop-mediated isothermal amplification; PCR, Polymerase Chain Reaction.

NOTE: LAMP represents the new HPV detection system based on LAMP and microfluidic chip. PCR represents DNA sequencing based on PCR methods.

**Supplementary Table 3.** **Comparison of test results of both LAMP and PCR & Sequencing in clinical samples infected with only one type of HPV**

| LAMP | PCR | | Total | Sensitive (%) | Specificity (%) | Kappa |
| --- | --- | --- | --- | --- | --- | --- |
|  | Positive | Negative |  | 95% CI | 95% CI |  |
| HPV27 | | | | | | |
| Positive | 12 | 7 | 19 | 100(73.54-100) | 61.11(35.75-82.70) | 0.557 (P=0.001) |
| Negative | 0 | 11 | 11 |  |  |  |
| Total | 12 | 18 | 30 |  |  |  |
| HPV2 | | | | | | |
| Positive | 1 | 3 | 4 | 100（2.5-100） | 89.66（72.65-97.81） | 0.366 (P=0.01） |
| Negative | 0 | 26 | 26 |  |  |  |
| Total | 1 | 29 | 30 |  |  |  |
| HPV1 | | | | | | |
| Positive | 5 | 0 | 5 | 100（47.82-100） | 100（86.28-100） | 1.000 (P<0.001) |
| Negative | 0 | 25 | 25 |  |  |  |
| Total | 5 | 25 | 30 |  |  |  |
| HPV57 | | | | | | |
| Positive | 1 | 0 | 1 | 100（2.5-100） | 100（88.06-100） | 1.000 (P<0.001) |
| Negative | 0 | 29 | 29 |  |  |  |
| Total | 1 | 29 | 30 |  |  |  |
| HPV7 | | | | | | |
| Positive | 0 | 1 | 1 | - | 96.67（82.78-99.92） | - |
| Negative | 0 | 29 | 29 |  |  |  |
| Total | 0 | 30 | 30 |  |  |  |

Abbreviations: HPV, human papillomaviruses; LAMP, Loop-mediated isothermal amplification; PCR, Polymerase Chain Reaction; CI, confidence interval.

NOTE: LAMP represents the new HPV detection system based on LAMP and microfluidic chip. PCR represents DNA sequencing based on PCR methods.
